# Distinct Subtypes of Apathy Revealed by the Apathy Motivation Index

**DOI:** 10.1371/journal.pone.0169938

**Published:** 2017-01-11

**Authors:** Yuen-Siang Ang, Patricia Lockwood, Matthew A. J. Apps, Kinan Muhammed, Masud Husain

**Affiliations:** 1 Nuffield Department of Clinical Neurosciences, University of Oxford, Oxford, United Kingdom; 2 Department of Experimental Psychology, University of Oxford, Oxford, United Kingdom; 3 Division of Psychology and Language Sciences, University College London, London, United Kingdom; Yeshiva University Albert Einstein College of Medicine, UNITED STATES

## Abstract

Apathy is a debilitating but poorly understood disorder characterized by a reduction in motivation. As well as being associated with several brain disorders, apathy is also prevalent in varying degrees in healthy people. Whilst many tools have been developed to assess levels of apathy in clinical disorders, surprisingly there are no measures of apathy suitable for healthy people. Moreover, although apathy is commonly comorbid with symptoms of depression, anhedonia and fatigue, how and why these symptoms are associated is unclear. Here we developed the Apathy-Motivation Index (AMI), a brief self-report index of apathy and motivation. Using exploratory factor analysis (in a sample of 505 people), and then confirmatory analysis (in a different set of 479 individuals), we identified subtypes of apathy in *behavioural*, *social* and *emotional* domains. Latent profile analyses showed four different profiles of apathy that were associated with varying levels of depression, anhedonia and fatigue. The AMI is a novel and reliable measure of individual differences in apathy and might provide a useful means of probing different mechanisms underlying sub-clinical lack of motivation in otherwise healthy individuals. Moreover, associations between apathy and comorbid states may be reflective of problems in different emotional, social and behavioural domains.

## Introduction

Apathy is a disorder of motivation characterised by reduced action initiation and goal-directed behaviour [1, 2]. Although it often occurs in several neurological and psychiatric disorders, it is also apparent to varying degrees in healthy people [3–9]. A lack of motivation can significantly affect everyday life, particularly in education and employment opportunities [10, 11]. Theoretical accounts have proposed that apathy is a multidimensional construct which actually covers motivation within dissociable domains: cognitive, emotional/affective and behavioural [2, 12]. Self-report and clinician administered measures have now been developed to characterise apathy in clinical samples based on this multidimensional construct (Lille Apathy Rating Scale [13], Dimensional Apathy Scale [14]). However, currently there are no validated assessments of apathy in healthy people. As a result, the mechanisms underlying variability in apathy are still poorly understood. It is also unknown whether different domains of apathy can be identified in healthy people, and whether they might be dissociable across individuals.

Whilst apathy is a common syndrome associated with altered motivation [2, 12], it is also frequently comorbid with other states which may have symptoms of reduced motivation, particularly depression, anhedonia and fatigue [3, 4, 15, 16]. This raises the question of the extent to which apathy can be meaningfully distinguished from these other conditions and whether they might perhaps be associated with discrete dimensions of apathy in healthy individuals.

In clinical disorders such as Parkinson’s disease (PD), it is now established that apathy is frequently linked to depression, with many overlapping symptoms including loss of interest and lack of initiative [17]. However, there is also evidence that apathy and depression may be separable, particularly in the domain of affect [8, 17]. Thus anhedonia, or loss in pleasure derived from activities one used to enjoy, is correlated with apathy in PD [15] and items used in the assessment of both symptoms are often overlapping [18, 19]. Recently fatigue—the feeling of exhaustion caused by the exertion of effort, which is unrelated to actual exertion of energy by muscles—has also been shown to associate with apathy in clinical disorders such as PD and multiple sclerosis [16, 20]. However, despite the evidence of links between apathy and depression, anhedonia and fatigue in neurological conditions, it remains to be established whether there are similar specific links in the healthy population. Furthermore, are these different symptoms associated with distinct profiles of apathy in healthy people?

Here, we adapted the Lille Apathy Rating Scale (LARS) [13], a tool first developed to measure apathy in PD, to produce and validate a novel measure to assess and dissect the profile of apathy in healthy people: the Apathy Motivation Index (AMI). Using the most rigorous psychometric procedures, we then dissected out the different factors that comprise the AMI and determined whether depression, anhedonia and fatigue are related to distinct profiles of apathy. We hypothesized that, after developing our new index, we would identify distinct subtypes of apathy in the general population and that these subtypes would be differentially associated with depression, anhedonia and fatigue.

## Study 1—Exploratory Factor Analysis

### Participants

505 people (211 males, 271 females, 23 gender undisclosed, mean age = 28.7 years, SD = 14.9, range = 16–85, N = 27 age undisclosed), recruited from the local communities via online adverts and posters, completed a preliminary 51-item scale. All participants gave written informed consent and the study was approved by the University of Oxford ethics committee. Methods were carried out in accordance with the relevant guidelines and regulations.

### Procedure

Our initial item validation was adapted from the LARS [13]. To create a comparable measure suitable for the general population, a team of clinical neurologists and university researchers developed, based on their experience with clinically apathetic patients, novel items to specifically reflect each domain of the LARS. Items from the clinical LARS that were deemed to be applicable to healthy people were also adapted. This gave rise to a preliminary 51-item scale [21]. Participants were asked to self-rate each item on a five-point Likert scale by deciding how true that statement was based on the past two weeks of their life. The scale ranged from 0–4 (with 0 = ‘completely untrue’, 4 = ‘completely true’). An “N/A” option was also available for items that were not applicable. Each item was reverse-scored so that a higher rating indicated more apathy.

### Data analysis

An exploratory factor analysis (EFA) with promax rotation was conducted in MPlus [22] to examine the latent structure of this 51-item apathy questionnaire. The exploratory-derived solutions were assessed by scree plot [23] and two absolute fit indices: Root Mean Square Error of Approximation (RMSEA) and Standardised Root Mean Square Residual (SRMR). A value of RMSEA and SRMR less than 0.08 is generally considered to be reasonable [24].

### Results

A simple three-factor structure was the most parsimonious account of the data. This structure had good model fit (RMSEA = 0.051 with 90% CI of 0.048–0.053, SRMR = 0.05) and was supported by scree plot [23], which showed the characteristic “elbow” or plateau in eigenvalues after 3 factors. After the EFA, twenty-one items were excluded, as their loadings were less than 0.40.

#### Item reduction

Next, the six highest loading items for each factor were retained to form a revised 18-item Apathy-Motivation Index (AMI) [25]. These three factors were labelled according to their common themes as (1) ***behavioural activation* (BA)**: tendency to self-initiate goal-directed behaviour (e.g. I get things done when they need to be done, without requiring reminders from others), (2) ***social motivation* (SM)**: level of engagement in social interactions (e.g. I start conversations without being prompted), and (3) ***emotional sensitivity* (ES)**: feelings of positive and negative affection (e.g. I feel awful if I say something insensitive). Each of the six questions for the three subscales is shown in Table 1. The factor loadings for each subscale were good (BA: 0.56–0.75; SM: 0.54–0.66; ES: 0.46–0.78).

**Table 1:** Apathy-Motivation Index (AMI, provided in S1 Appendix).

Item	Subscale	Statement
1	ES	I feel sad or upset when I hear bad news.
2	SM	I start conversations with random people.
3	SM	I enjoy doing things with people I have just met.
4	SM	I suggest activities for me and my friends to do.
5	BA	I make decisions firmly and without hesitation.
6	ES	After making a decision, I will wonder if I have made the wrong choice.
7	ES	Based on the last two weeks, I would say I care deeply about how my loved ones think of me.
8	SM	I go out with friends on a weekly basis.
9	BA	When I decide to do something, I am able to make an effort easily.
10	BA	I don't like to laze around.
11	BA	I get things done when they need to be done, without requiring reminders from others.
12	BA	When I decide to do something, I am motivated to see it through to the end.
13	ES	I feel awful if I say something insensitive.
14	SM	I start conversations without being prompted.
15	BA	When I have something I need to do, I do it straightaway so it is out of the way.
16	ES	I feel bad when I hear an acquaintance has an accident or illness.
17	SM	I enjoy choosing what to do from a range of activities.
18	ES	If I realise I have been unpleasant to someone, I will feel terribly guilty afterwards.

*Note*: BA = Behavioural Activation. SM = Social Motivation. ES = Emotional Sensitivity. Participants have to rate, based on the last two weeks how true each statement is (‘completely untrue’, ‘mostly untrue’, ‘neither true nor untrue’, ‘quite true’, or ‘completely true’). Each item is negatively scored such that a higher score indicates greater apathy (4 = ‘completely untrue’, 0 = ‘completely true’) and level of apathy is assessed by taking the mean rating of the items within the subscale.

## Study 2—Confirmatory factor analysis (CFA), construct validity and reliability of the AMI

### Participants

Data from a new group of 479 people recruited via online adverts and Prolific Academic (www.prolific.ac) was used for the analysis (for demographic information see Table 2). Exclusion criteria were self-reported neurological or psychiatric disorder. All participants gave electronic informed consent and the study was approved by the University of Oxford ethics committee. 63 of these participants also completed the AMI a second time between 6–8 days after initial completion to assess test-retest reliability.

**Table 2:** Participant characteristics.

Characteristic	
Age	Mean = 29.7 (10.7) years old, Median = 27.0, range 18–74 (N = 2 undisclosed)
Men: Women	230:249, (48.0% male)
Education level	Primary/ Elementary school (N = 1, 0.2%)
	Secondary/ Middle school (N = 33, 6.9%)
	Post-secondary non-tertiary education (N = 139, 29.0%)
	Bachelor’s Degree (N = 179, 37.4%)
	Master’s Degree (N = 101, 21.1%)
	Doctor of Philosophy (N = 26, 5.4%)
Employment	Student (N = 188, 39.2%)
	Full-time employed (N = 160, 33.4%)
	Part-time employed (N = 56, 11.7%)
	Self-employed (N = 29, 6.1%)
	Unemployed (N = 19, 4.0%)
	Housework at home (N = 10, 2.1%)
	Long-term disabled (N = 6, 1.3%)
	Retired (N = 11, 2.3%)

### Procedure

Participants completed the AMI to confirm the proposed three-factor structure. In addition, they were also asked to complete a set of established related measures to assess construct validity, noted below. Descriptive statistics of these additional measures are provided in Table 3.

**Table 3:** Descriptive statistics of related measures.

Measure	Mean	S.D.	Median	Range
**Apathy Evaluation Scale**				
Total	33.1	8.6	32	18–60
**Beck Depression Inventory**				
Total	11.2	10.2	8	0–53
**Dimensional Apathy Scale**				
Executive	9.3	4.7	9	0–23
Emotional	9.1	4.0	9	0–23
Behavioural/Cognitive	10.5	4.3	10	0–21
Total	28.9	9.2	28	3–61
**Snaith-Hamilton Pleasure Scale**				
Total	48.7	5.7	49	23–56
**Modified Fatigue Impact Scale**				
Physical	12.4	8.7	11	0–36
Cognitive	14.3	8.5	14	0–40
Psychosocial	3.0	2.2	3	0–8
Total	29.7	17.7	29	0–84

#### Apathy Evaluation Scale (AES) [12]

The AES is an 18-item scale that measures apathy as a single construct. Each item was scored on a 4-point Likert scale, with a higher total score indicating greater apathy (1–4: 1 = ‘very true’, 4 = ‘not true at all’ for positively scored items).

#### Dimensional Apathy Scale (DAS) [14]

The DAS is a 24-item scale that assesses apathy on three different subscales, namely executive, emotional and behavioural/cognitive initiation. Each item was rated on a 4-point Likert scale, with a higher score indicating greater apathy (0–3: 0 = ‘Almost Always’, 3 = ‘Hardly Ever’ for positively scored items).

#### Beck’s Depression Inventory (BDI) [26]

The BDI is 21-item scale that measures the severity of depression. Each item relates to a symptom of depression, e.g., hopelessness, and was scored on a 4-point Likert scale (0–3: 0 = least severe, 3 = most severe). A higher total score indicates greater depression.

#### Snaith Hamilton Pleasure Scale (SHAPS) [27]

The SHAPS is a 14-item scale that assesses hedonic tone, or ability to experience pleasure. While responses were made on a 4-point scale, for simplicity, Snaith et al. [27] scored each item in a binary manner (0–1: 0 = either ‘Strongly Agree’ or ‘Agree’, 1 = either ‘Strongly Disagree’ or ‘Disagree’). We followed Pluck and Brown [15] and Franken et al. [28] and scored responses using a 4-point Likert-style instead (1–4: 1 = ‘Strongly Disagree’, 4 = ‘Strongly Agree’) with higher scores reflecting greater hedonic tone. Conversely, lower scores indicated higher levels of anhedonia.

#### Modified Fatigue Impact Scale (MFIS) [29]

The MFIS is a 21-item scale that measures how fatigue affects daily life, with each item being rated on a 5-point Likert scale (0–4: 0 = ‘Never’, 4 = ‘Almost Always’). A higher score indicates a greater impact of fatigue on the individual.

### Data analysis

A confirmatory factor analysis (CFA) was conducted in MPlus [22]. Model fit was assessed using RMSEA, SRMR, and Comparative Fit Index (CFI). While a CFI of at least 0.90 is normally taken to indicate an acceptable model [24], it should be noted that this index calculates the fit difference between a null independence model (i.e. zero correlation between all observed variables) and the hypothesized model. This means that for a model with low item inter-correlations, the CFI may be lowered even if it describes the data adequately. Thus, it has been suggested that in the event of substantial low item inter-correlations, the CFI criterion could be relaxed to > 0.80 [30]. Approximately 65% of the inter-correlations in our data were low (< 0.20), hence, we adopted the relaxed CFI cut-off of 0.80 while ensuring that the cut-offs for RMSEA and SRMR were < 0.08.

### Results

**Fig 1:**
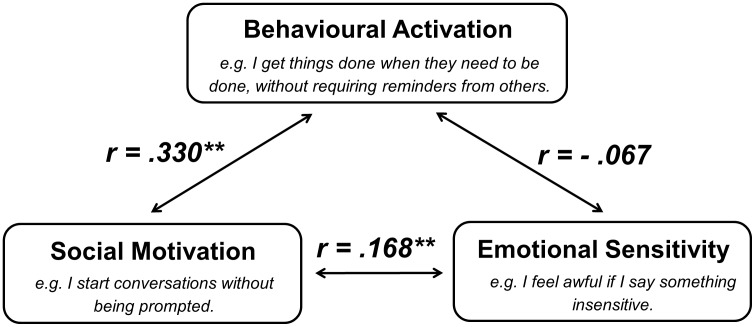
Apathy Motivation Index (AMI) factor model. The factor analysis identified three distinct subscales, namely behavioural activation (BA), social motivation (SM) and emotional sensitivity (ES). BA relates to an individual’s tendency to self-initiate goal-directed behaviour, SM examines a person’s engagement of social interactions and ES probes an individual’s feelings of positive and negative affection. While SM correlated with BA and ES, BA did not associate with ES. (**: *p* < 0.01).

The three-factor structure of the 18-item AMI (Table 1) was confirmed, and had good model fit indices (RMSEA = 0.076 with 90% CI of 0.068–0.083, SRMR = 0.071, CFI = 0.83). This model is schematically illustrated in Fig 1. Each item on the AMI was scored from 0–4, with a higher score indicating greater apathy. We propose cut-offs for moderate and severe apathy on the AMI to be respectively > 1 S.D. and > 2 S.D. above the mean (Table 4). While SM correlated significantly with BA and ES components of motivation, BA did not correlate significantly with ES. (**: *p* < 0.01).

**Table 4:** Proposed cut-offs for moderate (> 1S.D.) and severe (> 2S.D.) apathy on AMI.

AMI subscale	Mean (S.D.)	Proposed cut-off	
		Moderate	Severe
**Behavioural Activation**	1.58 (0.76)	≥ 2.34	≥ 3.10
**Social Motivation**	1.69 (0.74)	≥ 2.43	≥ 3.17
**Emotional Sensitivity**	1.05 (0.63)	≥ 1.68	≥ 2.31
**Total**	1.44 (0.47)	≥ 1.91	≥ 2.38

*Note*: Every AMI subscale consists of 6 items that is each scored from 0–4. Mean values and proposed cut-off scores for each subscale are given above. A higher mean rating indicates greater apathy on that subscale. Mean score based on ratings from 479 healthy people.

To assess internal reliability, Cronbach’s coefficient alpha values were calculated for both the total score and subscales. They showed adequate values, indicating acceptable internal consistency (α_overall_ = 0.77, α_BA_ = 0.79, α_SM_ = 0.75, α_ES_ = 0.75). Test-retest reliability coefficients for the scale and subscales were also satisfactory, indicating stable responses across time (r_overall_: 0.83, r_BA_: 0.88, r_SM_: 0.84, r_ES_: 0.72).

**Fig 2:**
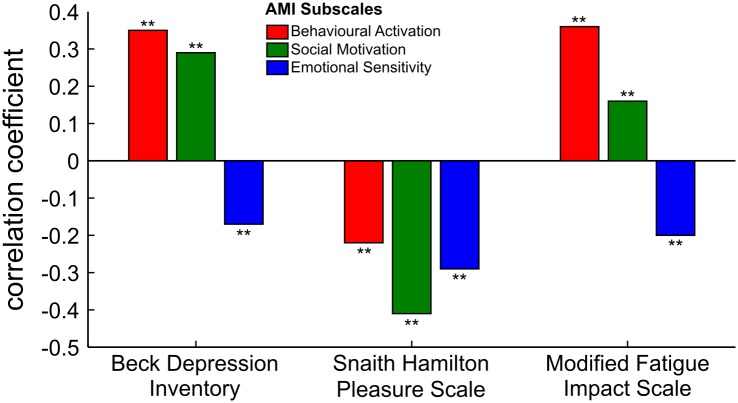
Correlation coefficients between AMI subscales and established measures of depression, anhedonia and fatigue. BA and SM correlated positively with BDI and MFIS, indicating that individuals that were more apathetic on these subscales also had higher levels of depression and fatigue. In contrast the ES scale was negatively correlated with depression and fatigue. All three AMI subscales were negatively correlated with the SHAPS (lower scores indicate higher levels of anhedonia), suggesting that higher apathy was associated with lower hedonia.

To examine construct validity, correlational analyses between the overall and subscale scores of the AMI and other related measures were conducted. The Benjamini and Hochberg method was used to control for false discovery on multiple comparisons [31]. We briefly summarize key correlational results here in text (details in Table 5 and Fig 2). AMI *total score* showed positive correlations with existing assessments of apathy: DAS total (*r* = 0.62, *p* < 0.01) and AES (*r* = 0.61, *p* < 0.01). It was also positively associated with the BDI (*ρ* = 0.26, *p* < 0.01) and MFIS total (*r* = 0.19, *p* < 0.01). Finally, the AMI total score was negatively correlated with the SHAPS (*r* = - 0.46, *p* < 0.01), indicating that apathetic people experience greater anhedonia. Overall, these findings indicated that the AMI had good construct validity.

**Table 5:** Relationship between AMI score and established measures of apathy (clinical indices), depression, anhedonia and fatigue.

	Apathy Motivation Index			
	Behavioural Activation	Social Motivation	Emotional Sensitivity	Total
**Dimensional Apathy Scale** ^a^				
Executive	**0.59** **	**0.14** **	**- 0.12** *	**0.34** **
Emotional	0.07	**0.22** **	**0.57** **	**0.41** **
Behavioural/Cognitive	**0.49** **	**0.53** **	0.08	**0.57** **
Total	**0.57** **	**0.42** **	**0.23** **	**0.62** **
**Apathy Evaluation Scale** ^a^				
Total	**0.55** **	**0.51** **	**0.11** *	**0.61** **
**Beck Depression Inventory** ^b^				
Total	**0.35** **	**0.29** **	**- 0.17** **	**0.26** **
**Snaith-Hamilton Pleasure Scale** ^a^				
Total	**- 0.22** **	**- 0.41** **	**- 0.29** **	**- 0.46** **
**Modified Fatigue Impact Scale** ^a^				
Physical	**0.26** **	**0.14** **	**- 0.19** **	**0.13** **
Cognitive	**0.41** **	**0.11** *	**- 0.20** **	**0.18** **
Psychosocial	**0.31** **	**0.31** **	**- 0.11** *	**0.28** **
Total	**0.36** **	**0.16** **	**- 0.20** **	**0.19** **

*Note*:

^a^ = Pearson correlation.

^b^ = Spearman correlation.

Correlations of *p* < 0.05 after correcting for multiple correlations are in bold.

* *p* < 0.05,

** *p* < 0.01.

## Study 3—Latent Profile Analysis (LPA): Classification of Apathy-Motivation Subtypes

### Participants

Participants were the same as in Study 2.

### Data analysis procedure

To examine whether we could identify distinct profiles of apathy and how these are differentially predictive of comorbid states (depression, anhedonia and fatigue), we conducted a latent profile analysis (LPA) [32] using the data acquired in study 2. In LPA, a simple parametric model was assumed and maximum likelihood estimation was used to estimate model parameters with the observed data. This allowed us to define the classes. Each individual’s probability of class membership was also estimated together with the overall model so that they can be placed into the appropriate class.

We investigated models with one to five classes and determined the optimal number of classes for our sample with several statistical indicators. The Lo-Mendell-Rubin Adjusted Likelihood Ratio Test [33] (LMRT) and Bootstrapped Likelihood Ratio Test (BLRT) [32] compares the fit of the current model with *K* classes to one with *K*-1 classes. A small *p*-value (< 0.05) indicates that the solution with *K* classes fit better. The Akaike Information Criterion (AIC) [34] and sample-size adjusted Bayesian Information Criterion (sBIC) [35] are descriptive fit indices with lower values indicating more optimal model fit.

### Results

The 4-class model was the most appropriate (Table 6). By iteratively comparing models one class apart (i.e. 1- versus 2-class, then 2- versus 3-class etc.) with the LMRT and BLRT *p*-values, we found the 4-class model had the best fit. Furthermore, the 4- and 5-class models had the lowest AIC and BIC values.

**Table 6:** Latent Profile Analysis (LPA) Model fit indices.

Number of Classes	1	2	3	4	5
**AIC**	3086	3020	3000	2968	2966
**sBIC**	3092	3030	3014	2986	2988
**LMRT *p*-value**	-	< 0.001	0.29	0.02	0.36
**BLRT *p*-value**	-	< 0.001	< 0.001	< 0.001	0.19

*Note*: AIC = Akaike Information Criterion, sBIC = sample-size adjusted Bayesian Information Criterion, LMRT = Lo-Mendell-Rubin Test, BLRT = Bootstrapped Likelihood Ratio Test. For AIC and sBIC, the smaller the value the more optimal is the model fit. For LMRT and BLRT of *K* classes, a small *p*-value (< 0.05) indicates that the solution with *K* classes fit better than that with *K*-1 classes.

**Fig 3:**
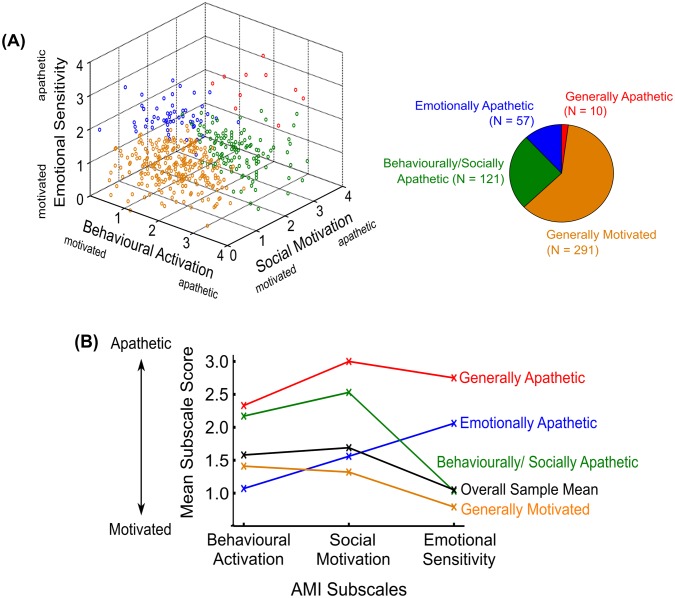
Distribution of apathy along the AMI subscales and conditional response means of the 4-class solution. The AMI consists of three subscales: Behavioural Activation, Social Motivation and Emotional Sensitivity. Every subscale contains 6 items that is each scored from 0–4, with a higher mean score indicating greater apathy. **(A)** 3D scatterplot illustrating the distribution of each healthy individual’s mean rating along the three AMI subscales. The four classes were labelled generally motivated (orange), behaviourally/socially apathetic (green), emotionally apathetic (blue), and generally apathetic (red). **(B)** Conditional response mean value greater than overall sample means (black line) indicates apathy on that AMI subscale.

To interpret each class, we compared the conditional response means with the overall sample means on each AMI subscale (Table 7, Fig 3). We then labelled these classes according to their profile on apathy subtypes:

- **Class 1** consisted of 57 individuals (11.9%) and was labelled **“emotionally apathetic”** as their mean ES subscale score was higher than that of the overall sample mean.
- **Class 2** contained 121 individuals (25.3%) and was labelled **“behaviourally/socially apathetic”** due to a higher mean BA and SM subscale score than the overall sample mean.
- All conditional response means for **Class 3** were lower than the overall sample means, thus, we referred to this class of 291 individuals (60.8%) as **“generally motivated”**.
- **Class 4** consisted 10 individuals (2.1%) who had substantially higher conditional response means than the overall sample means on every subscale. Accordingly, they were referred to as **“generally apathetic”**.

**Table 7:** Overall sample means and AMI profile conditional response means on the AMI Behavioural Activation, Social Motivation and Emotional Sensitivity subscales.

Subscale	Overall Sample Mean (*N* = 479) (S.D.)	Class 1: Emotionally Apathetic (*N* = 57) (S.D.)	Class 2: Behaviourally/ Socially Apathetic (*N* = 121) (S.D.)	Class 3: Generally Motivated (*N* = 291) (S.D.)	Class 4: Generally Apathetic (*N* = 10) (S.D.)
**Behavioural Activation**	1.58 (0.76)	1.07 (0.55)	2.17 (0.69)	1.41 (0.65)	2.33 (0.76)
**Social Motivation**	1.69 (0.74)	1.56 (0.57)	2.53 (0.47)	1.32 (0.49)	3.00 (0.54)
**Emotional Sensitivity**	1.05 (0.63)	2.06 (0.36)	1.03 (0.46)	0.79 (0.41)	2.75 (0.36)

*Note*: Every AMI subscale consists of 6 items that is each scored from 0–4. A higher mean rating indicates greater apathy on that subscale.

**Fig 4:**
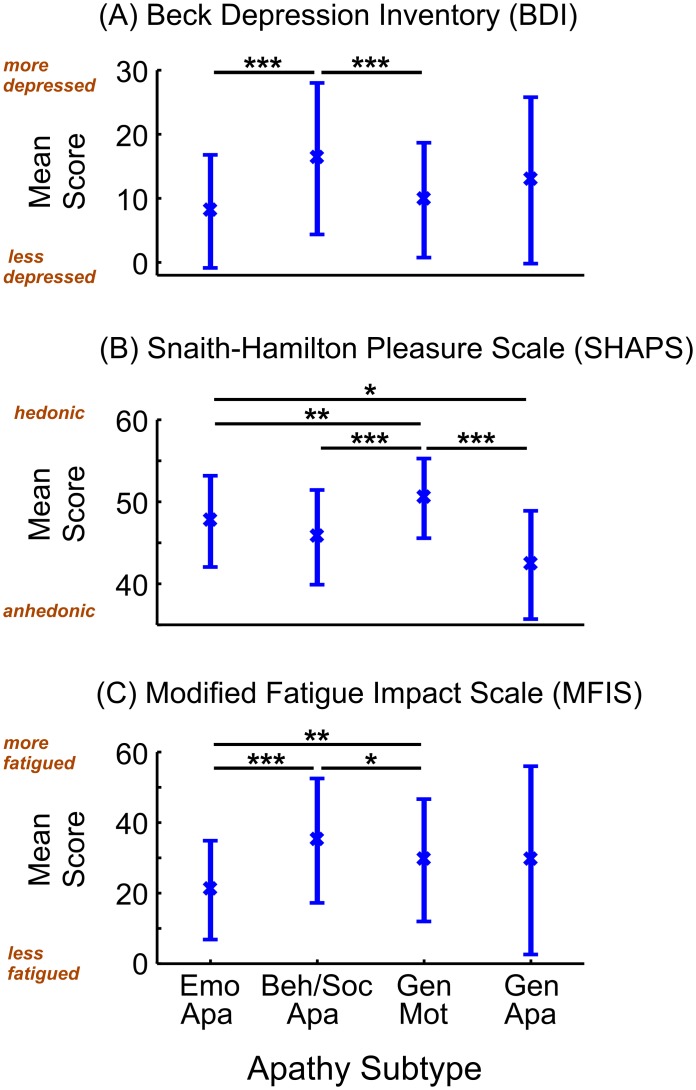
Relationships between apathy subtype and depression, anhedonia and fatigue. The four apathy-motivation subtypes were predictive of different associations with depression (A), anhedonia (B) and fatigue (C). The behaviourally/socially apathetic experienced the greatest depression and fatigue while the generally motivated were most hedonic. (* *p* < 0.05, ** *p* < 0.01, *** *p* < 0.001).

ANOVA was used to examine differences among the four apathy-motivation subtypes on independent measures of depression, anhedonia and fatigue (Fig 4). Significant differences were found between classes for all three measures (BDI: *F*(3,475) = 14.7, *p* < 0.001; SHAPS: *F*(3,475) = 29.9, *p* < 0.001; MFIS: *F*(3,475) = 8.63, *p* < 0.001).

Post-hoc comparisons showed that the behaviourally/socially apathetic class were significantly more depressed and fatigued than the emotionally apathetic and generally motivated classes. The generally motivated class also experienced greater fatigue than the emotionally apathetic class (Fig 4A and 4C). For anhedonia, the generally motivated class experienced more pleasure than each of the other classes. The emotionally apathetic people were also more hedonic than the generally apathetic class (Fig 4B).

All post-hoc comparison differences were significant at *p* < 0.05 (Emotionally apathetic: BDI mean = 8.0, SD = 8.8, SHAPS mean = 47.6, SD = 5.6, MFIS mean = 20.9, SD = 14.0; Behaviourally/socially apathetic: BDI mean = 16.2, SD = 11.8, SHAPS mean = 45.7, SD = 5.8, MFIS mean = 34.9, SD = 17.6; Generally motivated: BDI mean = 9.7, SD = 9.0, SHAPS mean = 50.4, SD = 4.9, MFIS mean = 29.3, SD = 17.3; Generally apathetic: BDI mean = 12.8, SD = 13.0, SHAPS mean = 42.3, SD = 6.6, MFIS mean = 29.3, SD = 26.7). Together, these results suggest that different subtypes of apathy are differentially predictive of depression, anhedonia and fatigue.

## Discussion

Here we developed the Apathy Motivation Index (AMI; Table 1), a new instrument suitable for assessing levels of apathy and motivation in the healthy population. Results of factor analyses—both exploratory (EFA) and confirmatory (CFA)–in large samples indicated that the AMI has a clear three-factor structure with good psychometric properties. We also showed for the first time that different subtypes of apathy are predictive of different associations with depression, anhedonia and fatigue in healthy people (Fig 4).

We identified three domains of apathy, namely behavioural activation (BA), emotional sensitivity (ES) and social motivation (SM). The BA subscale focused on the individual’s tendency to self-initiate goal-directed behaviour. This appears to relate closely to Stuss's [36] executive process of ‘energization’ or the initiating and maintaining of task-relevant responses. By contrast, the ES subscale contained items that probe an individual’s feelings of positive and negative affection, which seems similar to the emotional blunting often observed in some patients with apathy [37]. We also found that these two subscales were *not* significantly correlated, suggesting some dissociation between behavioural and emotional aspects of apathy.

Comparing these two subscales to the conceptualisation of apathy by Levy and Dubois [2] suggests that the BA subscale likely encompasses the ‘cognitive’ and ‘auto-activation’ aspects, while the ES domain appears to correspond more to the ‘emotional-affective’ subtype. This is supported by positive associations found between the AMI BA subscale with DAS (Dimensional Apathy Scale [14]) executive and behavioural/cognitive initiation but not the emotional subscale. Conversely, the AMI ES subscale was correlated with the DAS emotional subscale but not behavioural/cognitive initiation subscale.

The SM subscale contained items that examine a person’s engagement in social interactions. Impaired social life was highlighted by Sockeel et al. [13] as a domain of apathy during the development of the LARS, although it is not specified within other cognitive-behaviour-emotion frameworks of apathy [1, 2]. We observed that the AMI SM was correlated with both the BA and ES subscales. It also correlated positively with all three subscales of the DAS. This suggests that although separate factors of SM, BA and ES comprise apathy there is also some degree of shared variance between items assessing SM with BA and ES (Fig 1).

As expected, the AMI correlated with established assessments of apathy, depression, anhedonia and fatigue, providing evidence of good construct validity (Table 5). Rather than discuss every individual association between the scale/subscales, we highlight key findings here. Distinguishing apathy from depression is challenging due to the overlaps in symptoms, e.g., lack of initiation. Nonetheless, it is recognised that apathy may be characterised by emotional blunting whereas depression is an affective disorder featured by extreme emotional fluctuations [37]. In support of this, we observed that while the AMI BA and SM subscales correlated positively with the BDI, the ES subscale was negatively associated.

Anhedonia is a mood disorder characterised by an inability to derive pleasure. It has long been established that positive reinforcement is essential to maintain goal-directed behaviour [38]. Given this relationship between motivation and reward, we predicted that anhedonia and apathy would be linked. In support, we found that all subscales of the AMI were related to the SHAPS. Our finding suggests that apathy and anhedonia have a close relationship in the general population, with those individuals characterised by higher levels of apathy also more likely to report experiencing anhedonia. However, these measures were not perfectly correlated suggesting that there are also unique aspects of anhedonia not related to apathy.

Fatigue can be a symptom of reduced motivation characterised by the lack of energy to perform actions. Although similar in symptomology to apathy, few studies have investigated their relationship [16, 20]. We observed that the AMI BA and SM subscales associated positively with all subscales of the MFIS (namely physical, cognitive and psychosocial). Conversely, the ES subscale correlated negatively with these MFIS domains. These observations suggest that there is a partial overlap between apathy and fatigue. Specifically, while individuals who were behaviourally and/or socially more apathetic were likely to be more fatigued, people who were emotionally apathetic experienced less fatigue in general. Thus, there is also dissociation between fatigue and subtypes of apathy.

By using latent profile analysis (LPA) to group individuals with similar AMI profiles, we identified four subtypes of apathy-motivation in our healthy people (Table 7; Fig 3). Most people (60.8%) were identified as ‘generally motivated’ with their group average on each AMI subscale being lower than the overall mean. A small number (2.1%), on the other hand, scored higher than the global mean on all AMI subscales. Thus, these individuals were classified as ‘generally apathetic’. The other subtypes were labelled ‘emotionally apathetic’ (11.9%) and ‘behaviourally/socially apathetic’ (25.3%) as they had a lower average score than overall only on that/those particular AMI subscale(s).

Intriguingly, these different apathy subtypes were predictive of different associations with depression, anhedonia and fatigue. People identified as ‘behaviourally/socially apathetic’ were significantly more depressed and fatigued than people who were ‘emotionally apathetic’ or ‘generally motivated’. In addition, individuals who were ‘emotionally apathetic’, ‘behaviourally/socially apathetic’ or ‘generally apathetic’ were significantly more anhedonic than those who were ‘generally motivated’. These results indicated that the four apathy subtypes were distinct and overlapped differently with the closely related outcomes of depression, anhedonia and fatigue. Moreover, having high levels of behavioural and social apathy are more associated with other negative states, whereas experiencing emotional apathy on its own may not be associated with higher levels of depression and fatigue.

## Conclusion

The AMI is a reliable instrument suitable for assessing apathy and motivation in the healthy population and in clinical disorders. The findings presented here show for the first time that apathy in the healthy population can be dissected into four subtypes: ‘emotionally apathetic’, ‘behaviourally/socially apathetic’, ‘generally motivated’ and ‘generally apathetic’. These classes showed different propensities for depression, anhedonia and fatigue. Our data suggest that there may be particular subtypes of apathy that are more likely to co-occur with these symptoms. Future longitudinal studies would benefit from investigating how particular profiles of apathy are risk factors for the development of depression, anhedonia and fatigue.

## Supporting Information

S1 File File containing all data underlying findings for study 1 in the manuscript. (XLSX)

S2 File File containing all data underlying findings for study 2 in the manuscript. (XLSX)

S1 Appendix Apathy Motivation Index. (DOCX)
