# Supplementary material for: Distinct Subtypes of Apathy Revealed by the Apathy Motivation Index
Source: PLoS One. 2017 Jan 11;12(1):e0169938. doi: 10.1371/journal.pone.0169938 (PMC5226790; doi:10.1371/journal.pone.0169938)
Supplement: S1 Appendix — (DOCX) [file pone.0169938.s003.docx]

Below are a number of statements. Each statement asks you to think about your life over the last 2 weeks.

For each statement, select how appropriately it describes your life right now. Select “Completely true” if the statement describes you perfectly, “Completely untrue” if the statement does not describe you at all over the last 2 weeks, and use the answers in between accordingly.

| Completely UNTRUE | | Mostly untrue | | Neither true nor untrue | Quite true | | Completely TRUE | |  |
| --- | --- | --- | --- | --- | --- | --- | --- | --- | --- |
| 1 | I feel sad or upset when I hear bad news. | |  |  |  | |  |  | |
| 2 | I start conversations with random people. | |  |  |  | |  |  | |
| 3 | I enjoy doing things with people I have just met. | |  |  |  | |  |  | |
| 4 | I suggest activities for me and my friends to do. | |  |  |  | |  |  | |
| 5 | I make decisions firmly and without hesitation. | |  |  |  | |  |  | |
| 6 | After making a decision, I will wonder if I have made the wrong choice. | |  |  |  | |  |  | |
| 7 | Based on the last two weeks, I would say I care deeply about how my loved ones think of me. | |  |  |  | |  |  | |
| 8 | I go out with friends on a weekly basis. | |  |  |  | |  |  | |
| 9 | When I decide to do something, I am able to make an effort easily. | |  |  |  | |  |  | |
| 10 | I don't like to laze around. | |  |  |  | |  |  | |
| 11 | I get things done when they need to be done, without requiring reminders from others. | |  |  |  | |  |  | |
| 12 | When I decide to do something, I am motivated to see it through to the end. | |  |  |  | |  |  | |
| 13 | I feel awful if I say something insensitive. | |  |  |  | |  |  | |
| 14 | I start conversations without being prompted. | |  |  |  | |  |  | |
| 15 | When I have something I need to do, I do it straightaway so it is out of the way. | |  |  |  | |  |  | |
| 16 | I feel bad when I hear an acquaintance has an accident or illness. | |  |  |  | |  |  | |
| 17 | I enjoy choosing what to do from a range of activities. | |  |  |  | |  |  | |
| 18 | If I realise I have been unpleasant to someone, I will feel terribly guilty afterwards. | |  |  |  | |  |  | |

Scoring Instructions

Each item is negatively scored i.e. you will need to REVERSE ALL ITEMS:
Completely TRUE = 0
Quite true = 1
Neither true nor untrue = 2
Mostly untrue = 3
Completely UNTRUE = 4

Three domains of apathy-motivation are assessed with the mean score, which ranges from 0-4 (with 0 being motivated and 4 being apathetic).

1. Behavioural: Q5, 9, 10, 11, 12, 15
2. Social: Q2, 3, 4, 8, 14, 17
3. Emotional: Q1, 6, 7, 13, 16, 18
